# A tale of many tumors and one endocrine site: A case series of pituitary metastases

**DOI:** 10.5339/qmj.2021.38

**Published:** 2021-09-09

**Authors:** Zeinab Dabbous, Muna Mohamed, Silas Benjamin, Fiona Green, Hassoun Salman, Tarik Elhadd

**Affiliations:** ^1^Department of Medicine, Dumfries and Galloway Royal Infirmary, Dumfries, Scotland E-mail: zdabbous@hamad.qa; ^2^Endocrine Department, Hamad Medical Corporation, Doha, Qatar

**Keywords:** pituitary, panhypopituitarism, diabetes insipidus, metastatic breast cancer, metastatic lung cancer, endocrine tumors

## Abstract

Background: Metastases to the pituitary gland are extremely rare with an incidence rate reported from an autopsy series of 1.8%–12%, and only 20% was diagnosed clinically. Tumors that commonly metastasize are breast and lung tumors.

Case series: We present a series of five cases, including four female patients and one male patient with metastatic cancer. Two women had metastatic small lung cancer and presented with diabetes insipidus (DI). Two women had metastatic breast cancer, of which one presented with DI and the other with panhypopituitarism. The male patient had bronchogenic adenocarcinoma and presented with DI.

Conclusion: Our case series confirmed earlier reports that DI is the most common presentation of metastases to the pituitary gland.

## Background

Metastases to the pituitary gland are extremely rare events, and intracranial metastatic disease rarely involves the pituitary.^1^ Incidence rate reported from autopsy series ranged from 1.8% to 12%.^2,3^ Tumors that metastasize commonly to the pituitary gland are breast cancers and lung cancers,^4^ but any tumor may have metastatic potential, including hepatocellular carcinoma,^5^ thyroid carcinoma,^6^ and renal cell carcinoma.^7^

Diabetes insipidus is the main symptom correlating with the greater incidence of metastatic lesions in the posterior pituitary lobe.^2^ Other symptoms include headache, visual field deficit, and abnormal eye motility.^8^

Herein, we present five cases of pituitary metastasis from different centers and compare these cases with existing literature with regard to the primary tumor and presenting symptoms.

## Case Summaries

### Case 1

A 46-year-old woman was diagnosed with right breast cancer (infiltrating ductal carcinoma, locally advanced) in April 2016. She underwent staging by positron emission tomography (PET) which revealed bilateral axillary, supraclavicular, hilar, and mediastinal lymph node metastasis and uptake in the left breast, bone, and several liver metastases with intense uptake in the pituitary. She was started with goserlin, letrazole, and denosumab.

In June 2016, she started complaining of polydipsia. She was drinking more than 5 L of water per day. Magnetic resonance imaging (MRI) of the pituitary gland confirmed pituitary metastasis, and she exhibited signs and symptoms of pituitary insufficiency and diabetes insipidus. She was treated with suprasellar radiation therapy to the pituitary in July 2016 and maintained on replacement therapy mainly with levothyroxine, hydrocortisone, and desmopressin. She also had a small metastasis to the brain detected on MRI and was treated with gamma knife radiotherapy.

In September 2016, follow-up PET showed marked improvement with near resolution of bone uptake, resolution of hepatic uptake, and decrease uptake in both breast lesions.

In December 2016, repeated MRI of the brain showed re-demonstration of the previously depicted asymmetry of the pituitary gland being relatively and mildly more prominent on the right side with mild inclination of the pituitary stalk to the right. These findings are stable since the latest MRI. The previously depicted hyperintense small foci on tbl2 and fluid-attenuated inversion recovery sequences demonstrated a stationary course with no related edema, restricted diffusion, or abnormal enhancement.

Moreover, in December 2016, PET showed hypermetabolic lesion in the right thyroid lobe for further correlation. Moderate hypermetabolic soft tissue lesion was noted in the right breast and required further evaluation. Nonfluorodeoxyglucose-avid multiple sclerotic lesions were observed at multiple levels of the thoracic and lumbar vertebrae.

She maintained the same management, and follow-up imaging continued to show improvement. During this time, the patient was clinically well.

In January 2018, follow-up PET showed clear progression of the disease with progressing neck and mediastinal lymph nodes, as well as right breast lesion and new bone lesions. At this time, the oncologist decided to start second-line treatment for premenopause as ribociclib plus fulvestrant and to continue gosrelin and denosumab. She continued to receive replacement therapy for panhypopituitarism.

**Figure 1.**
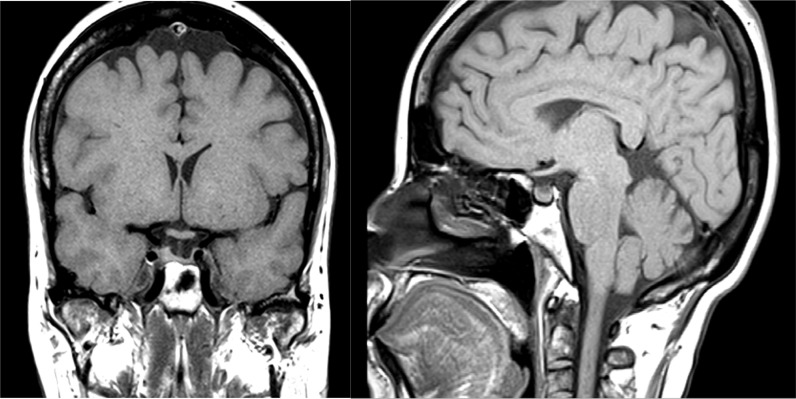
Case 1. Latest positron emission tomography scan showing complete metabolic resolution of the disease.

She responded well to treatment. In September 2018, the latest PET showed complete metabolic resolution of the disease (Figure 1).

### Case 2

An 80-year-old man was referred to the endocrinology clinic for headache, thirst, and polyuria of 4 months duration. He was known to have hypertension, diverticulosis, and vitamin B12 deficiency. He drank at least 5 L of fluids per day. He also lost 6 kg of weight. He had no other systemic or alarming symptoms.

On examination, he had normal visual fields and absence of cranial nerve palsy. He weighed 83.9 kg, and his body mass index was 28.4 kg/m^2^. Clinical examination was unremarkable, with no features suggestive of acromegaly, Cushing's, or any other endocrinopathies.

Results of baseline investigations were as follows: sodium of 147 mEq/L, plasma osmolality of 301 mosm/kg, prolactin of 1062 mU/L, and normal levels of other pituitary hormones.

The water deprivation test showed results consistent with cranial diabetes insipidus. At baseline, his urine osmolality was 65 mosm/kg; after 2 h of water deprivation, it was 319 mosm/kg; and after subcutaneous administration of desmopressin, it increased to 416 mosm/kg. He was started with desmopressin and demonstrated good therapeutic response.

**Figure 2.**
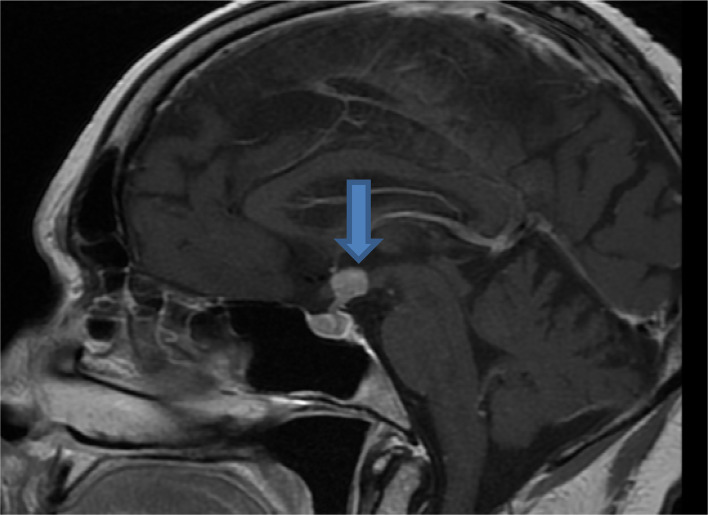
Case 2. Magnetic resonance image of the pituitary gland showing a mass lesion infiltrating the pituitary stalk.

MRI of the pituitary gland showed a 10-mm mixed cystic/soft tissue mass lesion filling the posterior aspect of the pituitary sella, with infiltration of the pituitary stalk (Figure 2). He continued to experience headache. Full-brain MRI confirmed the presence of a small nodule in each frontal lobe in addition to the pituitary mass lesion, likely metastatic deposits.

Computed tomography (CT) of the chest, abdomen, and pelvis showed a central tumor of the right lung (CT stage tbl4), prominent para-aortic and left hilar lymph nodes, and metastatic deposit in tbl11.

His general condition deteriorated very quickly, so he was not fit for palliative chemotherapy. He was referred to the palliative care team for symptom management. He died within 1 month of his diagnosis.

### Case 3

**Figure 3.**
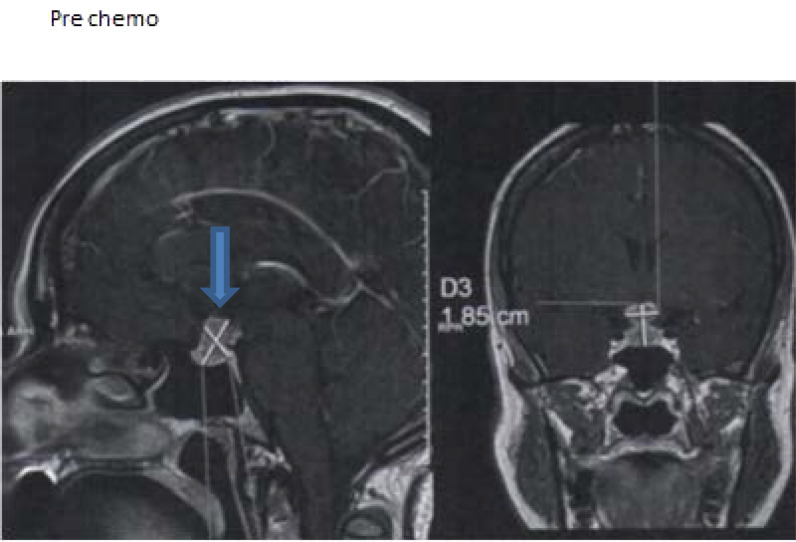
Case 3. Magnetic resonance image of the pituitary gland before chemotherapy.

A 42-year-old woman with a history of stage 4 breast cancer was referred from the oncology department for management of abnormal thyroid functions. In 2011, she was diagnosed with breast cancer, for which she underwent mastectomy, received chemotherapy, and discharged on tamoxifen. However, in 2016, she had recurrence of breast cancer and further received chemotherapy. Owing to severe back pain and headache, she had radiological imaging that showed spinal metastasis, and MRI of the pituitary gland detected a metastatic lesion (Figure 3). She also complained of polyuria and extreme tiredness. Initial investigations showed a low serum cortisol level and biochemical evidence of secondary hypothyroidism. She was started on replacement therapy.

In 2017, during clinic follow-up, she was taking hydrocortisone 25 mg daily, levothyroxine 75 mcg daily, and desmopressin 60 mcg once daily. She was very keen to reduce the dose and then stop hydrocortisone because she was gaining weight. Clinical examination on her first visit revealed a blood pressure of 115/76 and heart rate of 90 beats per min. She reported feeling tired at the end of the day.

Her biochemistry showed the following: serum cortisol (9:00) am of < 22 nmol/L, thyroid stimulating hormone (TSH) of 0.03 mU/L, free thyroxine of 9.93 pmol/L, and spot urine osmolality of 146 mosm/kg (50–1200) normal range.

**Figure 4.**
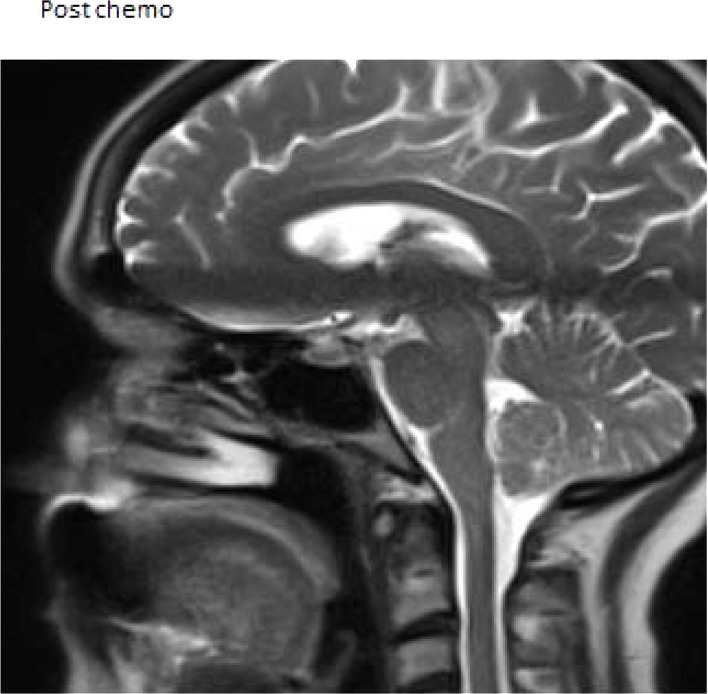
Case 3. Magnetic resonance image of the pituitary gland after chemotherapy showing an empty sella.

A repeat MRI of the pituitary gland after 8 months showed no metastasis or mass lesion but an empty pituitary sella (Figure 4).

Steroid replacement was optimized, and she maintained hydrocortisone 10 mg/5 mg/5 mg. She was also taking levothyroxine 150-mcg and desmopressin 60-mg tablets once daily. She is doing very well and still having denosumab injections six times weekly.

### Case 4

A 70-year-old woman with metastatic right breast cancer (spinal metastasis) and autoimmune hypothyroidism was referred to the endocrine clinic for thirst and polyuria. She was on prednisolone 10 mg to enhance weight and well-being and letrozole. The oncologist ruled out diabetes mellitus and hypercalcemia. Clinical examination was unremarkable.

Blood investigations revealed the following: normal sodium level, plasma osmolality of 296 mosm/kg, urine osmolality of 161 with low normal urine sodium, and elevated prolactin at 1051 mU/L. Cortisol was checked; however, it cannot be interpreted given her treatment with prednisolone. TSH was suppressed as she was on tbl3 replacement.

The water deprivation tests confirmed the diagnosis of cranial diabetes insipidus. At baseline, her urine osmolality was 161 mosm/kg; after 4 h of water deprivation, it was 288 mosm/kg; and after subcutaneous injection of desmopressin, it further increased to 609 mosm/kg.

She was started on Desmospray which immediately resolved her symptoms.

**Figure 5.**
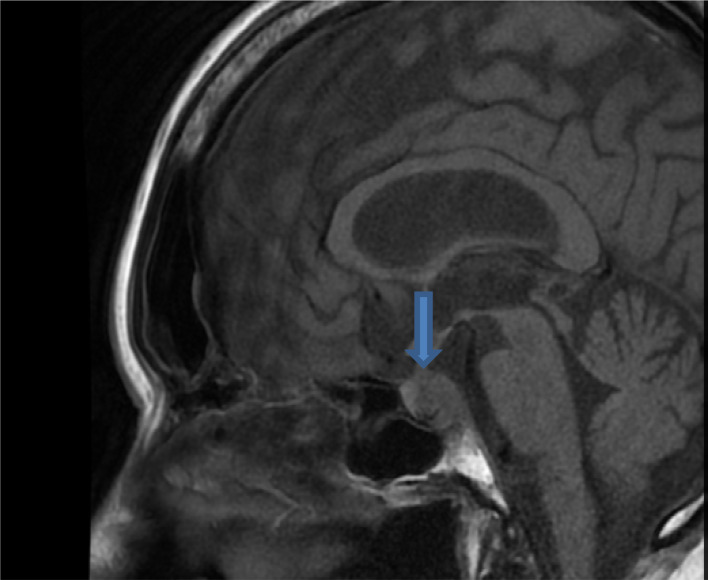
Case 4. Magnetic resonance image of the pituitary gland showing a pituitary stalk lesion.

MRI of the pituitary gland showed a 9-mm enhancing round lesion originating from the pituitary stalk impinging on the optic chiasm and optic tracts suggestive of metastasis (Figure 5).

She received radiotherapy to tbl9 and the whole brain but developed sepsis during her hospital stay. She died 8 months later.

### Case 5

A 50-year-old man presented to the hospital with a 3-week history of excessive thirst, urinary frequency, and right loin pain. He had felt generally unwell for the previous 8 weeks and had lost approximately 6 kg of weight over this time. He felt nauseous and had vomited several times. He urinated 4–5 times at night and had recorded a urine output of 3 L in 24 h at home. He had dull, right loin pain that did not radiate, was constant in nature, but exacerbated by movement. His appetite was grossly reduced. He was an ex-smoker [30 pack years].

On clinical examination, he was afebrile and normotensive. His heart rate was 70 beats per minute and regular.

Results on admission were as follows: plasma osmolality of 289 mosmol/kg, urine osmolality (random) of 396 mosmol/kg, and urine sodium of 23 mmol/L. He was admitted into the hospital, and during hospital stay, he underwent an endocrine profile, with results shown in Table 1.

His results were consistent with panhypopituitarism. He was commenced on hydrocortisone and subsequently felt much better. The nausea and vomiting stopped, and his 24-h urine output remained at 3 L.

He presented again to the emergency department 4 days after discharge with worsening nausea and vomiting. He was unable to eat and could not tolerate his prescribed medications. He was given fluids, hydrocortisone, and antiemetic intravenously. Within 48 h, he felt well and was able to eat and drink normally; therefore, IV fluids were stopped. The dose of hydrocortisone was doubled for another 48 h and given orally. Levothyroxine and testosterone were also started.

With further history assessment, he reported being unwell for more than a year, with excessive lethargy. He had decreased libido and was unable to maintain an erection.

Examination revealed cervical lymphadenopathy that had not been present or recorded on previous examination. An in-patient MRI of the head was performed and showed “multiple ring enhancing lesions [>50], including a lesion in relation to the pituitary stalk measuring 8 mm. There was no associated mass effect or significant edema” (Figure 5).

Chest X-ray imaging was performed and showed a left hilar mass. Urgent CT of the chest, abdomen, and pelvis was then arranged, which showed a large left upper lobe mass consistent with bronchogenic carcinoma. Lung, nodal, hepatic, and adrenal metastases were identified [CT staging tbl4, N3, M1]. Lymph node biopsy of a left cervical node was performed under ultrasound guidance. Histology confirmed a diagnosis of small cell carcinoma of the lung.

He was commenced on dexamethasone (hydrocortisone was stopped) along with thyroid hormone and testosterone, but he died 2 weeks later.

## Discussion

The five patients with pituitary metastasis presented herein are patients recalled from the clinics of each of the authors. Each patient comes from a different center; thus, the treatment approach for each case might be different.

Pituitary metastases are a relatively rare clinical condition. They occur in 1%–3.6% of patients with advanced cancer at autopsy.^3,9,10^ However, metastasis rates are as high as 27% in autopsy series that evaluated both the pituitary and surrounding sella turcica.^11^ Metastatic disease accounts for approximately 1% of all transphenoidal surgeries.^12^

Metastases are commonly caused by tumors in the posterior lobe of the pituitary, which is affected approximately twice as often as the anterior lobe.^3^ Teears and Sliverman reported that two-thirds of the lesions localized to the posterior pituitary, 13% to the anterior pituitary, and 12% to both lobes,^13^ and the remaining to the capsule or stalk.^14^

The mechanism of pituitary metastases is considered hematogenous, and the incidence of metastases is dominant in neurohypophysis in comparison with adenohypophysis. The most likely reason is that the posterior lobe receives blood directly from the systemic circulation, whereas the anterior part receives blood indirectly from the hypophysial portal system.^15^

Pituitary metastases appear to occur more frequently with certain types of tumors, such as breast and lung cancers that account for 50% and 20% of the cases, respectively,^4^ followed by prostate, renal, gastrointestinal, hematological, and thyroid malignancies. Our case series included two patients with breast cancer and three patients with lung cancer. Occasionally, no primary tumor can be identified.^16^ In some patients, symptoms due to pituitary metastases may be the first presenting feature of cancer.^17^ Actually, two of the patients presented with diabetes insipidus that led to pituitary metastasis diagnosis and then identification of the primary tumor.

Symptoms such as tiredness, fatigue, headache, weight loss, and vomiting, which are very common in advanced primary metastatic neoplasm, will mask anterior pituitary deficiency states.^14^ Higher prevalence rate of headache, diabetes insipidus, visual field defect, and cranial nerve palsy as presenting symptoms is reported in patients with a large metastatic sellar mass.^8^ In most case series, diabetes insipidus is reported as the most common symptom; its presence should cause a high clinical suspension to rule out metastatic sellar lesions.^18^ Treatment of anterior pituitary deficiency may unmask underlying diabetes Insipidus.^14^ Thyroid and adrenal deficiencies were the most common anterior pituitary manifestations of metastatic sellar lesions.^19^

Pituitary metastasis presenting with hormonal hyperfunction, i.e., acromegaly and Cushing's syndrome, is very rare.^20^ In our case series, four of five patients presented with diabetes insipidus and one patient had hypopituitarism.

The challenge lies in distinguishing a pituitary adenoma from a metastatic pituitary mass; this is important to avoid unnecessary therapeutic intervention. The presence of abducens nerve palsy, rapidity of clinical presentation, and diabetes insipidus, and a history of underlying primary neoplasm will point toward a metastatic pituitary lesion.^21^ Radiological evaluation of pituitary metastasis by CT or MRI showed that majority of the lesions are sellar or suprasellar enhancing mass in approximately 50% of the cases, followed by stalk enhancing or thickening lesions in 30%, and loss of high intensity signal in posterior lobe in approximately 15%.^20^

Metastatic pituitary has poor prognosis and will depend on the nature of primary neoplasia and its response to treatment. Endocrinologists should promptly recognize the hormone deficiency state and provide adequate hormone replacement therapy. A multidisciplinary approach is necessary in the management of metastatic pituitary lesions, involving an endocrinologist, oncologist, and neurosurgeon.

The vast majority of pituitary metastases remain undiagnosed, found incidentally or at autopsy, and are asymptomatic because the patients usually die from the complications of the primary disseminated cancer.^3,9,11^ However, early detection and appropriate treatment may improve the quality of life and even the prognosis.

## Conclusion

Our case series confirmed earlier reports that diabetes insipidus is the most common presentation of metastases to the pituitary gland. Herein, four of our five patients presented with such syndrome. Recent reports have shown that metastases may have a diverse source, including renal cell, follicular thyroid, and hepatocellular cancers, but lung and breast cancer are the leading ones.

### Funds

No funding

### Conflict of Interest

None declared.

### Conflict of interest

None declared.

The abstract was presented as a poster in the Endocrine Society Meeting 2019.

## Figures and Tables

**Table 1:** Results of endocrine profile

	Values	Normal range

TSH	0.57 mU/L	0.3–5.0 mU/L

FT4	5.9 pmol/L	11–24 pmol/L

FSH	< 0.5 U/L	

LH	< 0.1 U/L	

Total testosterone	< 0.6 nmol/L	8.7–27 nmol/L

Prolactin	684 mU/L	0– < 500 mU/L

Baseline cortisol level	40 nmol/L	133–537 nmol/L

Cortisol level 30 min after cosyntropin administration	489 nmol/L	< 550 nmol/L


TSH: thyroid stimulating hormone; FT4: free thyroxine; FSH: follicle-stimulating hormone; LH: luteinizing hormone.
